# Integrated Translatome and Proteome: Approach for Accurate Portraying of Widespread Multifunctional Aspects of *Trichoderma*

**DOI:** 10.3389/fmicb.2017.01602

**Published:** 2017-08-29

**Authors:** Vivek Sharma, Richa Salwan, P. N. Sharma, Arvind Gulati

**Affiliations:** ^1^Department of Plant Pathology, Choudhary Sarwan Kumar Himachal Pradesh Agricultural University Palampur, India; ^2^Department of Veterinary Microbiology, Choudhary Sarwan Kumar Himachal Pradesh Agricultural University Palampur, India; ^3^Institute of Himalayan Bioresource Technology Palampur, India

**Keywords:** transcripts, active mRNA, regulation, integrated omic, translatome

## Abstract

Genome-wide studies of transcripts expression help in systematic monitoring of genes and allow targeting of candidate genes for future research. In contrast to relatively stable genomic data, the expression of genes is dynamic and regulated both at time and space level at different level in. The variation in the rate of translation is specific for each protein. Both the inherent nature of an mRNA molecule to be translated and the external environmental stimuli can affect the efficiency of the translation process. In biocontrol agents (BCAs), the molecular response at translational level may represents noise-like response of absolute transcript level and an adaptive response to physiological and pathological situations representing subset of mRNAs population actively translated in a cell. The molecular responses of biocontrol are complex and involve multistage regulation of number of genes. The use of high-throughput techniques has led to rapid increase in volume of transcriptomics data of *Trichoderma*. In general, almost half of the variations of transcriptome and protein level are due to translational control. Thus, studies are required to integrate raw information from different “omics” approaches for accurate depiction of translational response of BCAs in interaction with plants and plant pathogens. The studies on translational status of only active mRNAs bridging with proteome data will help in accurate characterization of only a subset of mRNAs actively engaged in translation. This review highlights the associated bottlenecks and use of state-of-the-art procedures in addressing the gap to accelerate future accomplishment of biocontrol mechanisms.

## Introduction

*Trichoderma* is a cosmopolitan and cardinal representative soil microflora of various climatic conditions (Herrera-Estrella, 2014). The biocontrol role of *Trichoderma* spp. have emerged as an attractive choice in agriculture sector due to their environmentally friendly nature over synthetic pesticides (Mukherjee et al., 2012, 2013). Among different biocontrol agents (BCAs), the genus *Hypocrea*/*Trichoderma* containing *Trichoderma harzianum, Trichoderma atroviride, Hypocrea virens* are probably the most explored BCAs (Schuster and Schmoll, 2010; Sharma and Shanmugam, 2012; Sharma and Salwan, 2017) and occupies over 60% of all registered biopesticides (Mukherjee et al., 2013). The continuous efforts on the evaluation of biocontrol potential of *Trichoderma* have led to the identification of several promising species/strains including *T. harzianum* (Yedidia et al., 1999; Cloyd et al., 2007), *Trichoderma virens* (Hermosa et al., 2000; Howell, 2006), *Trichoderma viride* (Papavizas, 1985), *T. atroviride* (Longa et al., 2009), *Trichoderma polysporum* (Zhang et al., 2015), and *Trichoderma asperellum* GDFS1009 (Wu et al., 2017). In recent studies, another potential strains of *Trichoderma saturnisporum* has been identified for its biocontrol potential (Sharma and Shanmugam, 2012; Diánez Martínez et al., 2016). In addition to primary application in agriculture, *Hypocrea jecorina/Trichoderma reesei* strains are molecular factory for cellulolytic enzymes (Merino and Cherry, 2007; Singh et al., 2015). The natural potential to secrete lytic enzymes, antibiotics, and defeating opponent for space and nutrition are largely considered responsible for its success against plant pathogenic fungi (Viterbo et al., 2002; Benítez et al., 2004). The root colonization and intimate association of *Trichoderma* spp. with plant roots are known to promote plant growth and boost immune response against a number of plant pathogens (Contreras-Cornejo et al., 2011; Brotman et al., 2012; Mukherjee, 2012). Biocontrol strains of *Trichoderma* are used worldwide for the management of various plant pathogens like vascular wilt caused *Fusarium* (Al-Ani et al., 2013), Botrytis blight or gray mold caused by *Botrytis* (Elad and Kapat, 1999), anthracnose caused by *Colletotrichum* spp., and several other plant fungal diseases (Sharma et al., 2016a,b, 2017a). The improvement of *Trichoderma* species as BCAs for various agricultural applications required, detailed understanding of its active biological repertoire involved in mycoparasitism antibiosis as well as others components (**Table 1**). Genome sequencing and its annotation in mycoparasitic species have depicted genome sizes of 38.8 and 36.1 Mb for *T. viride* and of *T. atroviride* for biocontrol strains, respectively, compared to 34 Mb of *T. reesei* an industrial strain. Annotation of complete genome depicted a gene pool of 11,800 genes for *T. atroviride* and 12,400 genes for *T. viride*, compared to 9,143 genes in saprophytic strain *T. reesei*. The abundance of gene pool in mycoparasitic strains of *Trichoderma* genome (Lin et al., 2012; Atanasova et al., 2013) and expression of over 60% of the encoding transcripts during interaction of *T. virens* and *T. atroviride* against *Rhizoctonia solani* have revealed a complex nature of biocontrol mechanisms (Atanasova et al., 2013). Liu and Yang (2005) using simulated mycoparasitic conditions and cDNA libraries identified a total of 3,298 expressed sequence tags (ESTs) which corresponds to 1,740 transcripts. Using inducible conditions for *T. harzianum* CECT 2413, Vizcaíno et al. (2006) characterized, nearly 8,710 ESTs whereas Yao et al. (2013) identified 1,386 ESTs for *T. harzianum* 88. Among different ESTs, significant differential expression was observed only for limited transcripts. These EST represents a fragment of mRNA have several biotechnological applications and are being explored for either complementing the sequenced genome projects or cost effective alternatives for identification of genes as well as elucidation of functional genomics of plant–microbe interactions (Vieira et al., 2013). Advancement in molecular tools such as transcriptome profiling using RNA-seq and quantitative real-time PCR (RT-qPCR) technologies also predicted a large number of genes (14,095) for *T. harzianum* during augmentation on plant pathogen such as *Sclerotinia sclerotiorum* cell wall and only 297 were found differentially expressed among them (Steindorff et al., 2012, 2014). In addition to plant diseases management potential of biocontrol strains of *Trichoderma*, its growth promotion abilities in plants have been identified which are significantly enhanced during their antagonistic interactions with pathogens in soil. The molecular action of its biocontrol arsenal is mediated through adaptive recruitment and reprogramming of unique reservoir of several transcripts (Shaw et al., 2016). A comparative account using bioinformatic approaches such as BLAST analysis has revealed a very low overlap for different ESTs libraries (Yao et al., 2013). Therefore, the microarrays set designed based on genome coverage and ESTs may not provide accurate information.

**Table 1:** List of a few selected glycosyl hydrolases, secondary metabolites, and different transcripts of biocontrol strains/species of *Trichoderma* characterized for their role in biocontrol.

S. no.	Responsible biocontrol metabolites	Reference	Biological function
Lytic enzymes	**Chitinases** Endochitinases, chitobiosidase, *N*-acetyl-β-D-glucosaminidase	Faize et al., 2003; Hoell et al., 2005; Seidl et al., 2005; de las Mercedes Dana et al., 2006; Ike et al., 2006; Klemsdal et al., 2006; Lopez-Mondejar et al., 2009; Ihrmark et al., 2010; Gruber et al., 2011; Loc et al., 2011; Sharma and Shanmugam, 2012; Xie et al., 2014; Sharma et al., 2016a	• Targeted breakdown of pathogen’s cell wall through mycoparasitism • Induces resistance against biotic and abiotic stress responses • Root colonization
	**Glucanases-** Exo-β-D-(1,3/4/6)-glucanases, endo-β-D-(1,3/4/6)-glucanases	De la Cruz et al., 1995; Vazqez-Garciduen et al., 1998; De la Cruz and Llobell, 1999; Kulminskaya et al., 2001; Kim et al., 2002; Teresa et al., 2003; Nobe et al., 2004; Djonović et al., 2006; Grun et al., 2006; Montero et al., 2005, 2007; Xie et al., 2014	
	**Proteases**	Elad and Kapat, 1999; De Marco and Felix, 2002; Szekeres et al., 2004; Viterbo et al., 2004; Simkovi et al., 2008; Atanasova et al., 2013; Dou et al., 2014; Sharma et al., 2016b, 2017a; Wu et al., 2017	
	**Amylase**	de Azevedo et al., 2000	
Secondary metabolites	**Antibiotics** Gliotoxin, viridin, gliovirin, glisoprenin, hepteledic acid. **VOCs and other metabolites** 6-Pentyl-α-pyrone, hydrocarbons, alcohols, ketones, aldehydes, alkanes, alkenes, esters, aromatic compounds, heterocyclic compounds, and terpenes, koninginins, anthraquinones, trichodermamides, polyketides, terpenoids, trichodermaides, azaphilones and harzialactones, massoilactone, glisoprenins, heptelidic acid, etc.	Reino et al., 2008; Zhang et al., 2014; Garnica-vergara et al., 2015; Kottb et al., 2015; Bae et al., 2016; Chen et al., 2016; Lee et al., 2016; Zeilinger et al., 2016	• Antimicrobial action • Plant defense stimulator • Mycoparasitism/competition • Root morphogenesis • Promote plant growth and growth regulator changes
	**AMPs** Non-ribosomal-derived antimicrobial peptides such as peptaibols, harzianic acid, Trichopolyn VI, alamethicins, harzianin HA V and saturnisporin SA IV, etc.	Garo et al., 2003; Viterbo et al., 2007; Maischak et al., 2010; Shi et al., 2012, 2016; Panizel et al., 2013; Röhrich et al., 2015	• Antimicrobial and insecticidal • Induces plant resistance • Inhibition of primary roots • Programmed cell death • Electrical long distance signaling in plants
	**Siderophores and organic acids** Phenolate type, organic acids such as gluconic, citric, or fumaric acid	Anke et al., 1991; Qi and Zhao, 2013	• Mineral acquisition through chelation and acidification of soil
Root colonization	**Hydrophobins**	Sanna, 2006; Espino-rammer et al., 2013; Ruocco et al., 2015; Przylucka et al., 2017	• Biotic and abiotic stresses • *Trichoderma*–plant–pathogen three-way interaction • Potential role in stimulating the activity of cutinases on poly(ethylene terephthalate)
Miscellaneous	**Transporters**	Ruocco et al., 2009; Reithner et al., 2011; Shentu et al., 2014; Steindorff et al., 2014; Morán-Diez et al., 2015; Sharma et al., 2017a,b	• Tolerance to phytotoxins/and their detoxification

The comprehensive analyses using different molecular approaches including ESTs (Chambergo et al., 2002), subtractive hybridization (Carpenter et al., 2005; Scherm et al., 2009; Vieira et al., 2013), microarray (Chambergo et al., 2002; Breakspear and Momany, 2007; Samolski et al., 2009), and transcriptomes (Atanasova et al., 2013) have established the complex response of *Trichoderma* species in biocontrol process which induces numerous genes having morphogenetic or other functions as well (Mehrabi-Koushki et al., 2012; Puglisi et al., 2012; Cacciola et al., 2015; Cetz-Chel et al., 2016). The complexity in different attributes may not be related to a particular stress and hence can lead to either imperfect transcriptional representation or a complex response. The continuous development in molecular technologies and advent of cloning free libraries using genome sequencing, deep RNA sequencing and proteomics has played vital role in the accurate identification and enhancing our capabilities of cataloging mRNA and protein populations exclusive to *Trichoderma* strains in response to changing environmental conditions (Shentu et al., 2014; Xie et al., 2015; Schmoll et al., 2016).

The *Trichoderma*–plant–pathogen interaction can produce significant amount of noise. Therefore one can speculate that the substantial amount of response at the gene expression level represents noise and that only a few changes are adaptive. Also, the microbes in the environment are continuously subjected to challenges and respond simultaneously to these factors in a complex way. Understanding the regulatory interactions necessitates an approach that can encompasses simultaneous both the transcriptome and proteome to observe and systematically view the adaptive expression at RNA and protein level. The integrated studies based on translatome and proteome level can provide a better state of these adaptive responses during biocontrol interaction. The regulation of mRNA at transcriptional and post-transcriptional levels contributes to reprogramming the behavior of BCAs through protein and secondary bioactive metabolites secretion to counter the pathogen associated challenges.

**FIGURE 1:**
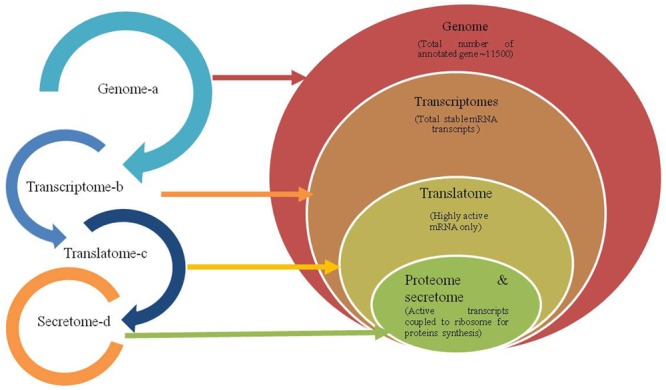
Schematic flow of genetic information from genome to proteome level. A- Relationship of different omic approaches in *Trichoderma*–plant–plant pathogen interaction. B- Hierarchal relationship of total gene predicted using stacked ven diagram in the genome to the total mRNA transcribed (transcriptome) representing both stable and highly active mRNAs population under a stress response. Here depending upon the conditions and alternate splicing a > b > c > d or a > b > c = d or rarely a >b = c=d; a > b = c < d.

So far studies on *Trichoderma* have been conducted extensively using ESTs and transcriptome approach revealed the expression of several genes related to mycoparasitism of BCAs directly (Reithner et al., 2011; Sharma et al., 2017b) or indirectly through the modulation of host transcriptome (Morán-Diez et al., 2012; Perazzolli et al., 2012). In our previous studies, attempts were made to identify the role of different transcripts related to lytic enzymes, transporter system, and other gene related to metabolites of *T. harzianum* (Sharma et al., 2016a,b, 2017b) and characterization of extracellular proteins from *T. saturnisporum* (Sharma and Shanmugam, 2012) using autoclaved mycelium of different plant pathogenic fungi. These studies revealed only a limited number of proteins compared to transcripts. The approaches used for cDNA cloning and other array technologies have also created artifacts in accurate identification of candidate transcripts. Therefore, the integrated translatome and proteome based studies can help in a better and accurate depiction of key regulators involved in *Trichoderma*–plant–pathogen interaction (**Figure 1**). Recent studies showed that the gene expression of mycoparasitic *T. harzianum* and *T. atroviride* strains changes not only to plant-pathogenic fungi (Sharma et al., 2016a, 2017b) but also to itself (Reithner et al., 2011). Thus translational response is a key determinant contributing to adaptation under such interaction stress (Picard et al., 2013). Therefore, present review emphasizes the role of translatome based approach in accurate determination of active mRNA population in a complex dialog coupled to proteome data in a three way interaction of *Trichoderma*–plant–pathogen.

## Mechanisms of *Trichoderma*

**FIGURE 2:**
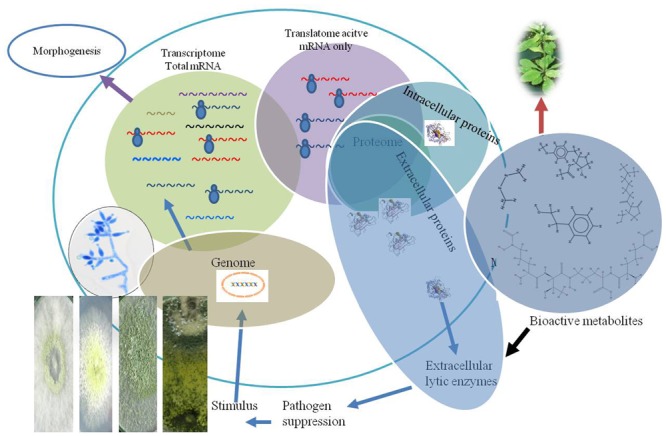
Pictorial representation of total mRNA transcripts, active mRNA involved in protein and bioactive metabolites synthesis during interaction with plant or plant pathogens. The figure explains that transcriptome based study in general represents a higher level of mRNA, compared to total translatome which represents only ribosomal loaded active mRNAs during interaction.

*Trichoderma* strains are used as BCAs in agriculture largely due to their abilities to directly antagonize plant-pathogenic fungi through the production of hydrolases (Benítez et al., 2004; Gruber and Seidl-Seiboth, 2012), antibiotics (Rubio et al., 2009; Vinale et al., 2014) and their tolerance to toxin produced by plant pathogens (Sharma et al., 2013) (**Table 1**). The interaction of *Trichoderma* with host plants reprograms not only the gene expression of biocontrol strains but also of its associated host plant (Harman, 2011; **Figure 2**). For example, strains of *Trichoderma* are explored for growth promotion and boosting immune responses, root development, and activation of seed germination or amelioration of abiotic stresses (Harman et al., 2004; Lorito et al., 2010; Shoresh et al., 2010; Hermosa et al., 2012). The immune responses in host plant are primed through systemic resistance (Tucci et al., 2011), involving a complex signaling of jasmonic acid/ethylene-induced systemic resistance and/or salicylic acid-dependent pathways which may behave differently in plant–*Trichoderma* interactions (Shoresh et al., 2010). The three way interaction between biocontrol, host plant, and pathogen from initial root colonization is known to change both the transcripts and proteome of host plants (Alfano et al., 2007; Segarra et al., 2007; Shoresh and Harman, 2008; Palmieri et al., 2012; Gomes et al., 2017; Martínez-Medina et al., 2017a,b; Pelagio-Flores et al., 2017). The availability of microarrays, next generation DNA sequencing, RNA-seq, and genome annotation have provided a global insight into the transcriptome response of plant–*Trichoderma* and *Trichoderma*–plant pathogen interaction.

## Omics Approaches in Uncoupling Genome and Transcriptome Profile

The characterization of candidate transcripts involved in various biological functions using transcriptome is one of the best approach. In comparison to stable nature of the genome, transcriptome is more dynamic and vary in response to different stimuli. The massive transcriptome response to various factors can be tentatively identified, quantified, and correlated to a biological process using ESTs, subtractive libraries, and DNA microarrays (Herrera-Estrella, 2014). A number of studies have been done at genome-wide and transcriptional level to understand the molecular behavior of different *Trichoderma* strains under contrasting conditions ranging from mycoparasitism of plant pathogens to imparting direct beneficial aspects to plants under stress conditions (Arvas et al., 2006; Vizcaíno et al., 2007; Seidl et al., 2009). The transcriptome analysis of *T. atroviride* IMI206040 at different stages of interaction with *R. solani* identified 7,797 out of 11,863 estimated genes which represented over 65% of total gene of the organism genome whereas only 1.47% of total gene (175) transcripts were found significantly differentially expressed in mycoparasitic interactions. The differentially expressed transcripts were also investigated during pathogenic attack on *Phytophthora capsici, Botrytis cinerea*, and *R. solani* (Reithner et al., 2011). In comparison to a large number of transcribed genes predicted for *T. atroviride* based on genomic data, only 38.4% of genes involved in interaction with *R. solani*, were expressed before contact whereas 52.8% were found responsible for *Trichoderma* confrontation with itself (Reithner et al., 2011).

The use of EST (Vizcaíno et al., 2006, 2007), subtractive cDNA libraries and DNA array (Rosales-Saavedra et al., 2006; Alfano et al., 2007; Mathys et al., 2012) based studies carried under environmental conditions have helped dramatically to the global-scale identification of active genes of *Trichoderma* which are not directly linked to plant pathogens but are required for colonization and imparting other beneficial aspects to the host plant. For example, hydrophobins, aspartyl proteases, expansin-like protein of *Trichoderma* origin have been explored for their involvement in the mycoparasitism mediated biocontrol of these microbes (Brotman et al., 2008; Samolski et al., 2009). Subsequently, the sequencing of complete genome and high-throughput transcriptome using 454 sequencing (Barakat et al., 2009) has enhanced our understanding on investigation of mechanisms at global cellular level under different conditions in better way (Reithner et al., 2011). The transcriptome based approach is far more robust, dynamic, and refined technique compared to genome sequencing which is stable as described below.

### *Trichoderma* Genome Organization

Since the genome sequencing of *T. reesei* industrial strain nine years back (Martinez et al., 2008), presently the genome of a number of strains representing *T. virens, T. harzianum, T. atroviride, T. asperellum, Trichoderma longibrachiatum*, and *Trichoderma citrinoviride* have been sequenced and revised (http://genome.jgi.doe.gov/). A comparative account of genome revealed presence of seven chromosomes in industrial strain *T. reesei* (Carter et al., 1992; Mantyla et al., 1992; Herrera-Estrella et al., 1993) whereas six chromosomes in biocontrol strains *T. harzianum* and *T. viride* (Gómez et al., 1997; Martinez et al., 2008). The genomic annotation of *T. virens, T. atroviride*, and *T. reesei* also unveiled lack of transposons and remarkable similarity of genes up to 78–96% among them. In the genome of *T. virens* and *T. atroviride* no true orthodox were reported for 2,756 and 2,510 genes, respectively in other species. The genome of *T. virens* and *T. atroviride* share 1,273 exclusive orthologs and 26 expanded families which were missing in *T. reesei* genome that may be a probable answer to mycoparasitic nature of *T. atroviride* and *T. virens* (Kubicek et al., 2011; Herrera-Estrella, 2014). A comparative study of genome organization of two *Trichoderma* species has revealed the expansion of considerable expansion genes involved in mycoparasitic *T. virens* strain which are missing in *T. reesei* (Kubicek et al., 2011).

### Transcriptome

The development of modern sophisticated omics technologies has played a vital role in developing better system-level understanding of gene expression. In particular, transcriptome based studies have proved a yardstick in the investigation of global cellular mechanisms and identification of several key genes involved in mycoparasitism and imparting other benefits to the host by *Trichoderma* strains. The measurement of the entire set of RNAs through transcriptome coupled with DNA microarrays or high-throughput RNA sequencing is a reliable and reproducible tool for wide analysis of transcripts. A number of transcriptome studies have been done on *Trichoderma*–plant–pathogen interaction (Marra et al., 2006; Chacon et al., 2007; Samolski et al., 2009; Mehrabi-Koushki et al., 2012; Rubio et al., 2012).

Stating from initial use of EST for the determination of glucose metabolism in *T. reesei* (Chambergo et al., 2002) and TrichoEST project (Vizcaíno et al., 2006), ESTs based studies have been done in *T. harzianum* (Liu and Yang, 2005; Vizcaíno et al., 2006; Suárez et al., 2007; Yao et al., 2013), *T. atroviride, T. asperellum* (Vizcaíno et al., 2007; Liu et al., 2010), *T. virens* (Vizcaíno et al., 2007; Morán-Diez et al., 2010), *Trichoderma aggressivum, T. viride*, and *T. longibrachiatum* (Vizcaíno et al., 2007) for the identification of transcripts induced during mycoparasitism and other environmental conditions. From a total of unique sequences (3,478), in *T. harzianum* CECT2413, 23% were found related to secretory chitinases, glucanases, and proteases. A large number of transcripts expressed (9478 ESTs containing 2,734 unique sequences) during the early interaction of *T. atroviride* with *B. cinerea* and *R. solani* were identified (Seidl et al., 2009) whereas 66 genes covering 442 ESTs were induced under mycoparasitic interaction (Herrera-Estrella, 2014).

Similarly, the analysis of transcriptomics changes in *T. harzianum, T. virens*, and *T. hamatum* during interactions with tomato plants revealed expression of 1,077 genes and only six of them being common to all three. The majority of genes encoding enzymes belong to chitin degradation during early interactions with tomato plants whereas genes encoding other secreted proteins were likely to involve in the signaling between *Trichoderma* and plants. Transcriptome based studies have led to the identification of new candidate genes having role in redox reaction, possible elicitors, transporters (Sharma et al., 2017b), lipid metabolism and detoxification (Chacon et al., 2007; Sharma et al., 2013), small secreted proteins (Ruocco et al., 2009; Samolski et al., 2009; Rubio et al., 2014). The *de novo* sequencing of *T. atroviride* IMI206040 transcriptome obtained during mycoparasitic interaction in presence of plant-pathogenic fungus *R. solani* revealed thousands of high-quality reads. An account of transcripts expressed during interaction to the total number of genes predicted in the genome of *T. atroviride* revealed that almost 45% were induced during interaction with *R. solani* and only 175 of them were host responsive (Reithner et al., 2011; Gupta et al., 2016).

Microarray analysis of *T. harzianum* T34 strain interaction with *Arabidopsis* identified approximately 24,000 transcripts of the host plant which were modulated by the BCA. The significance and global impact of this beneficial microbe in reprogramming the molecular physiology of host plant to stress responses through the regulation of transcription, signal transduction pathways has been reported in different studies (Morán-Diez et al., 2012; Lamdan et al., 2015). Further host specific response of *Trichoderma* strain with plants representing monocot and dicot hosts under the same conditions have also been explored to identify signature transcriptome repertoires and answer the widely prevalent questions of specificity of responses and role of secreted proteins in mutualistic interaction, root colonization, and induction of immune responses (Morán-Diez et al., 2015; Ho et al., 2016; Sharma et al., 2017b). These studies indicate the limitations of transcriptome based studies in precise estimation of ribosome loaded active mRNA population involved in complex mycoparasitic behavior of *Trichoderma* species as BCAs.

### Translatomes

The mRNA and protein levels do not perfectly correlate in native or engineered systems (Tian et al., 2004; Jayapal et al., 2008; Vogel and Marcotte, 2012; Payne, 2015). The post-transcriptional regulation of transcripts is a complex process and may not be compared with transcription level regulation of genes. Therefore, the post-transcriptional regulation is of great significance for better characterization of functional role of genes (Picard et al., 2013). Although ESTs and transcriptome based experimental studies have provided valuable information in mining genes incited by various stress responses in *Trichoderma* interaction with plants and plant pathogens, its application is limited because the levels of the proteins and their encoding mRNA are not correlated to each other. Therefore considering the use of the cutoff standards in transcriptome based studies and appearance of artifacts in the differential expression of genes, translatome based studies offers potential choice and a better alternative involving only active mRNA populations (Picard et al., 2013; Yanguez et al., 2013; Piccirillo Ciriaco et al., 2014; King and Gerber, 2016; Meteignier et al., 2017).

**FIGURE 3:**
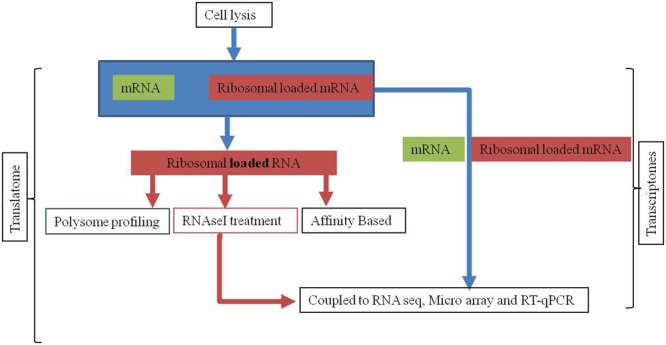
The experimental procedure of transcriptome and translatome for genome wide studies. In translatome ribosomal loaded or protected fragments of RNA are size or affinity fractionated, recovered, and ligated to adaptors for reverse transcription, amplification, and high-throughput RNA-seq whereas in transcriptome complete mRNA is used for subsequent analysis.

Studies involving translational regulation of gene expression are emerging as a prominent tool for the understanding the regulation of protein abundance in adaptive responses of the host (Halbeisen and Gerber, 2009; Spriggs et al., 2010). In the genetic flow of information, the translational regulation reprograms the cell activities by protein synthesis. In last decade due to rapid advancements in technology, efforts on understanding the modulatory role of translation in gene expression have increased significantly. The translatome referring to the active mRNAs population associated with ribosomes has facilitated the removal of background noise and useful for the accurate determination of active mRNA. Originally used in oocytes and embryos (Terman, 1970; Gurdon et al., 1971), translational control has emerged as a key point of eukaryotes. The process is executed by loading of ribosomes on mRNA followed by translation elongation (Groppo and Richter, 2009; Jackson et al., 2010). Since, the translatome based studies are focused only on the pools of genome-wide translated mRNA and therefore have helped in identification of key regulatory factors that are under translational control (Zupanic et al., 2013). This technique offers immense potential in the targeting key regulators which are active during interaction and play important role for the host plant in combating various stress responses. Translatome studies also help in determination of the ribosome number on active mRNA molecule in response to stress in the cellular genes (Koritzinsky and Wouters, 2007; Thomas and Johannes, 2007; Picard et al., 2013; **Figure 3**).

Presently, there are three methods used for translatome analysis; (a) polysomal profiling, (b) ribosomal profiling, and (c) ribosome affinity purification (RAP) (**Figure 3**). Polysomal profiling discovered in 1960s involves the separation of actively translated mRNAs bound by several ribosomes from free RNA by sucrose gradient centrifugation and then mRNAs can be coupled to northern blot or RT-qPCR or cDNA microarrays, or RNA-seq on a global level (Karginov and Hannon, 2013; Spangenberg et al., 2013). The second method known as ribosomal profiling was developed by Weissman group in *Saccharomyces cerevisiae*, determines the location of ribosomes at codon or nucleotide scale (Ingolia et al., 2009). The advantage of this technique is acquisition of information at global scale with respect to the position of the ribosomes on translated mRNA.

The deep nucleotide sequencing of ribosome protected RNA fragments obtained after RNase I treatment of cell lysate helps in accurate determination of ribosome position and its densities along RNA (Ingolia et al., 2012). Both polysome and ribosome based profiling need relatively large sample size to obtain enough RNA for microarray/RNA-seq analysis. The third method known as RAP developed by Inada et al. (2002) in *S. cerevisiae* capture monosomes and polysomes by using antiFLAG affinity resin. The RAP also known as translating RAP provides a better approximation of the translated mRNA population if coupled with transcriptome analysis (Halbeisen and Gerber, 2009; Jiao and Meyerowitz, 2010).

### Integrating Translatome and Proteomic Study

The post-transcriptional events such as translation regulation and protein stability are the principle causes of weak correlations and variations in proteomic, transcriptomic, and genomic data. The associated errors in transcriptome analysis are subjected to arise from the suppression by microarrays which can further impede the identification of active candidate transcripts. On the other side, methods opted for protein staining, limitations associated in visualizing low-abundant and co-migrating proteins seriously hampers proteomic based study. The recent developments in proteomics methods such as use of mass spectrometric (MS) and liquid chromatography (LC) techniques have made quantitative proteomic profiling, currently a driving force for identification of proteins. The highly stable and reproducible performance of mass spectrometers such as Q Exactive hybrid quadrupole-Orbitrap mass spectrometer MS and Triple TOF 5600 MS is capable of identification of both proteomics (Chang et al., 2014) and characterization of bioactive metabolites. Integrated analyses of active mRNAs coupled with protein expression are available for bacteria, yeast, mice, and humans. Similar to transcriptome, the translatome based studies are focused only on transcripts level which are intracellular in nature. The coupling of multiomic approaches based on active mRNA, proteomes, and protein turnover of both intra as well extracellular proteins and biologically active metabolites under different environmental conditions will provide a better answer of reprogramming biocontrol to various plant beneficial attribute and its resiliencies to combat different environmental conditions (**Figure 2**).

## Conclusion

The availability of the fully sequenced genomes of *Trichoderma* spp. has accelerated our research on understanding of the behavior of different species of this genus and how the information on their gene pool determines their capabilities and limitations. The genomes of *Trichoderma* which is known to contain thousands of genes encoding different glycosyl hydrolases, secondary metabolites, antibiotics, lectins with insecticidal properties, and transporters with potential in bioremediation involved in antibiotics biosynthesis, and several other candidate genes (Druzhinina et al., 2012; Atanasova et al., 2013). Exploration of genes and their encoding proteins involved in developing tolerance against various stresses such as cold, below-average precipitation, salty conditions, pH, herbicide resistance as well biotic factor are an active field of research. The predicted genome of *Trichoderma* strains are known to encode a large number genes therefore coupling of translatome studies with proteomics of both extracellular and intracellular proteins offers a wide scope for better understanding the complex behaviors of *Trichoderma* as BCA.

The genomic comparison of mycoparasitic species of *T. harzianum* with non-mycoparasitic strains of *T. reesei* already provides evidences of the expansion of several genes in biocontrol strains. The secretion of a large number of cell wall targeting enzymes and bioactive secondary metabolites require adaptive molecular reprogramming of *Trichoderma* transcriptome. The variation at genomic, transcriptomics, and proteomic levels is a challenging task and difficult to correlate due to complex and non-systematic post-transcriptional and limitation of proteomic techniques. Further, the translational control is a widespread phenomenon with intense effect; nevertheless it is underestimated for its regulatory roles. In general, extensive uncoupling of both RNA movements and inferred cell activities has been observed for 19 different transcriptome and translatome. Therefore, coupled quantitative transcript and protein abundance studies can serve as a gold standard for proper and accurate depiction of interaction involving *Trichoderma*–plant–plant pathogens. Although detecting changes in the transcriptome level (total mRNAs), translatome level (ribosome loaded mRNAs) and the proteome is experimentally feasible in a high-throughput way, the integration of these omic technologies is still far away. Systematic global analyses aims at integrating transcriptome, translatome, and proteome level can provide accurate view of widespread adaptive mechanisms of interaction between *Trichoderma*–plant–pathogen.

In future, integrated efforts will help us to better understand, identify, and then explore the molecular behavior of *Trichoderma* arsenal involved in its success as BCAs as well as industrial sectors. In such instances, the integration of the translatome using ribosomal profiling and coupling it with proteomic approaches such as liquid chromatography-tandem mass spectrometry (LC-MS/MS) for both extracellular and intracellular proteins offers a lot of scope for accurate characterization of active molecular components involved in biocontrol and then subsequently their utilization of various applications.

## Future Directions

A comparative multiomic coupled insights of *Trichoderma*–plant–plant pathogens in three way interaction will play vital role in accurate characterization of transcripts responsible for cosmopolitan nature of *Trichoderma* and then targeting the promising one for agricultural based applications. The latest advancements and complete genome sequencing have already provided a platform of gene pool. Further integration with latest functional techniques such as translatome will lead another step close to identification of targets in the form of active transcripts involved in a complex interaction of plant–BCA–plant pathogens.

## Author Contributions

VS and RS prepared the manuscript. PS and AG edited the manuscript.

## Conflict of Interest Statement

The authors declare that the research was conducted in the absence of any commercial or financial relationships that could be construed as a potential conflict of interest.
